# Factors associated with culture proven neonatal sepsis in the Ho municipality 2016

**DOI:** 10.11604/pamj.2020.36.281.20408

**Published:** 2020-08-14

**Authors:** Fortress Yayra Aku, Patricia Akweongo, Kofi Mensah Nyarko, Lord Graceful Mensah, Kokou Amegan-Aho, Lawrence Kumi, Edwin Andrew Afari, Donne Kofi Ameme, Ernest Kenu

**Affiliations:** 1 Ghana Field Epidemiology and Laboratory Training Programme, Department of Epidemiology and Disease Control, School of Public Health, University of Ghana, Legon, Ghana,; 2 School of Public Health, University of Health and Allied Sciences, Hohoe, Ghana,; 3 Namibia Field Epidemiology Training Programme, Ministry of Health and Social Services, Wndhoek, Namibia,; 4 Regional Health Directorate, Ghana Health Service, Volta Region, Ho, Ghana,; 5 School of Medicine, University of Health and Allied Sciences, Ho, Ghana

**Keywords:** Neonatal sepsis, antenatal clinic, parity, culture, Ho municipality

## Abstract

**Introduction:**

Neonatal Sepsis (NNS) is a public health problem which causes death or disability unless appropriate antibiotic treatment is given promptly. Globally, sepsis is an important cause of morbidity and mortality in neonates despite recent progress in health care delivery. We assessed the factors associated with culture proven sepsis among neonates in the Ho Municipality, Ghana.

**Methods:**

a cross-sectional study was conducted in two public hospitals in the Ho Municipality between January and May, 2016. All neonates who were clinically suspected with sepsis in the Neonatal Intensive Care Unit (NICU) and their mothers were recruited. A 2ml blood sample was taken aseptically and dispensed into a mixture of thioglycollate and tryptone soy broth in a 1: 10 dilution and microbiological procedures performed. Case notes of both neonates and their mothers were reviewed and interviews conducted to collect both clinical and socio-demographic data. We determined the factors associated with culture proven neonatal sepsis using logistic regression model and statistical significance was determined at 95% confidence intervals.

**Results:**

out of 150 neonates, 26 (17%) had laboratory confirmed sepsis. The most common pathogen isolated was Staphylococcus epidermidis 14, (54%). Neonates whose mothers were primigravida (OR=2.74; 95% CI: 1.12-6.68), and those who attended antenatal clinics (ANC) fewer than three schedules (OR=2.90; 95% CI: 1.06-7.96) had higher odds of developing culture proven sepsis.

**Conclusion:**

neonates who were the first babies of their mothers were more likely to develop laboratory confirmed sepsis. Also, neonates of mothers who attended ANC less than 3 times were more likely to develop laboratory confirmed sepsis. High index of suspicion is required to diagnose neonatal sepsis among neonates of primigravida mothers and mothers who attend fewer than three ANC schedules.

## Introduction

Neonatal Sepsis (NS) refers to a clinical syndrome that is marked by signs and symptoms of infection in the first 28 days of life, with or without isolation of a pathogen [1]. Globally, sepsis is an important cause of morbidity and mortality in neonates despite recent progress in health care delivery [2]. Over 40% of under-five deaths occur worldwide among neonates, leading to 3.1 million neonatal deaths annually. These deaths disproportionately occur in low-income countries and approximately one million of the deaths are linked to infectious causes including neonatal sepsis, meningitis, and pneumonia [3]. When neonates develop sepsis within 72 hours of life, it is termed as Early Onset Sepsis (EOS); and Late Onset Sepsis (LOS) if it develops after 72 hours of life [4]. Generally, the mother's colonized genitourinary tract is the source of infection for EOS and the related organisms include Group B Streptococci, Escherichia coli, Coagulasenegative Staphylococci, Haemophilus influenzae,and Listeria monocytogenes.The microorganisms in LOS are horizontally acquired and include Coagulasenegative Staphylococci, Staphylococcus aureus, Escherichia coli, Klebsiella species, Pseudomonas species, Candida species, Acinetobacter speciesand anaerobes[5]. Hence EOS and LOS are attributed to different maternal or hospital acquired organisms, with the latter usually associated with higher probability of antibiotic resistance [6]. Several risk factors have been identified to predispose neonates to sepsis and have been found to be linked to pregnancy, delivery practices, or other neonatal diseases [7]. Some maternal factors include maternal fever, instrumental delivery, and foul smelling liquor [8]. Low birth weight and prematurity are among the predisposing neonatal factors reported [9]. Other studies have also associated neonatal sepsis with low Apgar score in the fifth minute, and place of delivery. Strategies and processes that lead to the reduction of neonatal sepsis have a bearing on identifying and understanding of these factors. Therefore, identifying these factors in health care settings and providing timely interventions can reduce sepsis related morbidity and mortality. We the1ore sought to determine the factors associated with culture proven sepsis in two major hospitals in the Ho Municipality of the Volta Region, Ghana.

## Methods

**Study design and setting:** the study was a cross-sectional study conducted at two public hospitals in the Ho Municipality of the Volta Region of Ghana between January and May, 2016. The study population was neonates admitted at the Neonatal Intensive Care Units (NICU) of the Volta Regional and Ho Municipal Hospitals. Both hospitals are the two main public health care facilities in the municipality; with the regional hospital serving as a main referral centre. There are health care staff including clinicians and nurses that manage the NICUs.

**Inclusion and exclusion criteria:** all neonates that were admitted at the NICUs of both hospitals, who were clinically diagnosed of sepsis by a clinician during the study period, and whose mothers or caretakers consented to be part of the study were included in the study. However, neonates who met the inclusion criteria but died immediately before blood culture sample could be obtained, or those who were referred to a tertiary facility immediately upon assessment were excluded.

**Sample size and sampling method:** a prevalence of 11% of neonates with sepsis, with a 5% margin of error to obtain a normal deviate at 95% confidence level was used to calculate the minimum sample size of 150. All neonates that met the inclusion criteria and whose mothers or caretakers consented to be part of the study within the period were serially recruited until the sample size was obtained.

**Data collection:** a structured questionnaire was used to collect socio-demographic, clinical, and laboratory data on the neonates. Mothers of neonates who were recruited were interviewed to obtain their socio-demographic data. Case notes of both neonates and their mothers were reviewed to collect clinical data.

**Sample collection and laboratory investigation:** the antecubital fossa of neonates was cleaned twice with 70% alcohol and veins located. Trained laboratory scientists obtained 2ml blood samples aseptically from neonates into culture bottles containing a mixture of thioglycollate and tryptone soy broth in a 1: 10 dilution, labelled and transported into the laboratory for microbiological procedures to be performed on them. Samples were incubated overnight at 37°C then, sub-cultured unto commercially prepared blood, chocolate and MacConkey agar. The sub-cultured agars were incubated overnight at 37°C under both aerobic and anaerobic conditions and observed for growth. Agars with significant growth were identified for specific pathogens. Samples with no growth were incubated and observed for 7 consecutive days before determined as negative for culture. Data were then collected on the causative organisms that were isolated.

**Data management and analysis:** all variables collected were given unique identifiers and entered into Microsoft excel software. Data analysis was done using STATA software version 13.0. Continuous variables were presented as means and standard deviation whiles categorical variables were presented in tables as frequencies and proportions. Binary logistic regression was used to determine the association between culture proven sepsis and maternal sociodemographic; neonatal and pregnancy related factors. Variables that had a p-value <0.05 were entered into a multiple into a logistic regression model in a forward stepwise direction. The level of significance was set at 95% confidence interval.

**Ethical issues:** approval for this study was obtained from the Ethical Review Committee of the Research and Development division of the Ghana Health Service (GHS-ERC09/10/15). Permission was obtained from the Volta Regional Health Directorate, the management teams of both hospitals, as well as the management teams of the Neonatal Intensive Care Units (NICU) of the participating hospitals. Informed consent was sought from mothers or caretakers of neonates before recruiting them into the study. Each study participant was given a unique identifier to ensure confidentiality. All data collected were also kept under lock and key, such that no unauthorized person had access to them except the principal investigator.

## Results

**Neonatal characteristics:** of the 150 neonates that were recruited for the study, 91 (60.7%) were males. The majority of the mothers, 87 (58%) delivered by caesarean section and the rest by spontaneous vaginal delivery. The median APGAR score at one minute was 6 (IQR=6-8). Majority of the deliveries 109, (72.7%) were within the health facilities.

**Pregnancy related characteristics:** the age of mothers to the recruited neonates ranged from 16 to 41 years with a mean of 28 ± 6 years. Majority, 59 (39.3%) of mothers of the recruited had junior secondary education as their highest level of education. It was followed by 35, (23.3%) who had senior secondary education as the highest level of education. Of the 150 mothers of neonates recruited, 28, (18.7%) were unemployed.

**Common isolates identified:** of the 150 samples cultured, 26 showed culture-proven sepsis. There were equal proportions of causative organisms identified in both early and late onset sepsis. The gram positive organisms isolated were *Staphylococcus epidermidis* and *Staphylococcus aureus*. Four gram negative organisms were identified: *Pseudomonas aeruginosa, Escherichia coli, Enterobacter species* and *Proteus mirabilis*. The most common organism isolated was *Staphylococcus epidermidis*, 14 (53.9%) (Table 1). Of the 150 samples cultured, 26 showed culture-proven sepsis. There were equal proportions of causative organisms identified in both early and late onset sepsis.

**Table 1:** distribution of isolates of blood culture of neonates with sepsis, Ho municipality, 2016

Isolate	Early Onset Sepsis		Late Onset Sepsis		Total Count (N) (%)
	Count	Percentage (%)	Count	Percentage (%)	
Gram Positive organism					
Staphylococcus epidermidis	9	69.2	5	38.5	14 (53.9)
Staphylococcus aureus	1	7.7	3	23.1	4 (15.4)
Gram Negative organism					
Escherichia coli	1	7.7	0	0	1(3.8)
Pseudomonas aeruginosa	2	15.4	2	15.4	4 (15.4)
Enterobacter species	0	0	2	15.4	2 (7.7)
Proteus mirabilis	0	0	1	7.7	1(3.8)
Proteus mirab					
**Total**	13		13		26 (100.0)

**Factors associated with culture proven sepsis:** neonates of mothers who were employed had the odds of 0.55 (95% CI: 0.21-1.48) of developing neonatal sepsis whiles neonates whose mothers were married had the odds of 0.56 (95% CI: 0.23-1.35) of developing neonatal sepsis (Table 2)… Neonates who were delivered outside the study facilities had the odds of 2.29 of developing culture proven sepsis (95% CI: 0.95-5.54). The odds of developing culture proven sepsis by neonates on whom mechanical ventilation was performed was 1.64 (OR=1.64; 95% CI: 0.31-8.61) (Table 2(suite))…

**Table 2:** maternal socio-demographic factors associated with culture proven sepsis, Ho municipality, 2016

Variable	Sepsis	No sepsis	Crude Odds Ratio	95% CI	p-value
**Maternal age**					0.77
≤20	3	17	1		
21-30	14	57	1.39	0.36 - 5.42	
≥31	9	50	1.02	0.25 - 4.21	
**Marital status**					0.2
Married	16	92			
Single	10	31	1		
Divorced	0	1	0.56	0.23 - 1.35	
**Educational level**					0.53
No formal education	1	11			
Primary	4	14	1		
JSS	8	51	3.14	0.31 - 32.28	
SSS	6	29	1.73	0.20 - 15.24	
Tertiary	7	19	2.28	0.25 - 21.12	
**Employment**					0.25
Employed	7	22	1		
Unemployed	19	102	0.55	0.21 - 1.48	

* JSS: Junior secondary school; SSS: senior secondary school

**Table 2(suite):** maternal socio-demographic factors associated with culture proven sepsis, Ho municipality, 2016

Variable	Sepsis	No sepsis	Crude Odds Ratio	95% CI	p-value
**Sex**					0.92
Male	16	75	1		
Female	10	49	0.96	0.40 - 2.28	
**Birth weight**					0.85
<2500g	6	29	1		
≥2500g	20	95	0.9	0.33 - 2.50	
**Place of birth**					0.07
Within study facilities	15	94	1		
Outside study facilities	11	30	2.29	0.95 - 5.54	
**Gestational age (weeks)**					0.09
≤36	6	35	1		
37-40	14	80	1.02	0.36 - 2.88	
≥41	6	9	3.89	1.01 - 14.97	
**Length of hospital stay**					0.25
≤7days	16	61	1		
≥8days	10	63	0.61	0.25 - 1.44	

Neonates of mothers who were primiparous and mothers who attended antenatal clinic three times or less had the odds of 2.81 (95% CI: 1.17-6.74) and 3.0 (95% CI: 1.12-8.03) respectively of developing culture proven neonatal sepsis (Table 3). In a multivariate logistic analysis, gravidity and the number of antenatal visits of a mother showed significant association with culture proven sepsis. The odds of neonates whose mothers were primigravida having culture proven sepsis was 2.74 higher compared to neonates whose mothers had multiple gravidity (OR=2.74; 95% CI: 1.12-6.68). The odds of neonates whose mothers attended antenatal clinic ≤3 times having culture proven sepsis was 2.9 higher, compared to neonates whose mothers attended antenatal clinic ≥4 times (OR=2.90; 95% CI: 1.06-7.96) (Table 3)…

**Table 3:** pregnancy related factors associated with culture proven sepsis, Ho municipality, 2016

Variable	Sepsis	No sepsis	Crude Odds Ratio	p-value	95% CI	Adjusted Odds Ratio	95% CI	p-value
**Parity**				0.054		-		
Multiparous	11	78	1			-		
Primiparous	15	46	2.31		0.98 - 5.46	-		
**Gravidity**				0.02				
Multigravida	14	95	1			1		
Primigravida	12	29	2.81		1.17 - 6.74	2.74	1.12 - 6.68	0.03
**Maternal fever**				0.95		-		
No	24	113	1			-		
Yes	2	10	0.95		0.20 - 4.62	-		
**Number of antenatal visits**				0.04		1		
≥4	18	108	1			1	1.06 - 7.96	0.04
≤3	8	16	3		1.12 - 8.03	2.9		
**Delivery mode**								
SVD	13	50	1	0.37		-		
C/S	13	74	0.68		0.29 - 1.58	-		

## Discussion

Findings in this study revealed 17.3% of laboratory confirmed neonatal sepsis, out of the 150 neonates that were recruited based on clinical signs and symptoms. This was slightly lower than the 21.8% that was found in a similar study conducted in Uganda [10]. This could have been a result of the finding that 41 (27.3%) of neonates were born outside the study facilities, where there was no laboratory capacity for blood culture and hence administration of antibiotics in these neonates before referral before could have influenced positivity of blood culture in this study. The commonest pathogen isolated in this study was Gram positive organisms, 18 (69%). This finding corroborates with similar studies conducted in Dhakar, where 51.5% of Gram positive organisms were obtained of a total of 88 positive blood culture in a NICU.

The socio-demographic factors of mothers to neonates including age, education and employment had no significant association of neonates having culture proven sepsis. In contrast, a similar study showed that rates of prematurity and low birth weight, which are risk factors to neonatal sepsis are associated with the socioeconomic status of the mothers [11]. Multiple logistic regression reveals that, neonates whose mothers attended antenatal clinic fewer than 3 times have a higher odds of having culture proven sepsis (2.9) compared to neonates whose mothers attended at least four antenatal clinic. This suggests that mothers who attended fewer than three antenatal clinics may not have received sufficient education on the healthy practices to adopt to prevent transmission of infection to the neonates. In addition, these mothers stood a lower chance of getting screened for any infections that could lead to sepsis in the neonates due to less contact times with the health care providers. Though a similar study in Uganda looked at antenatal clinic times, they reported a higher odds (1.47) of culture proven sepsis among mothers who attended antenatal clinic fewer than 3 times compared to mothers who attended antenatal clinic more than 3 times [10].

Neonates whose mothers were primigravidous showed greater odds of developing culture proven sepsis after adjusting for number of antenatal clinic attendance. Pregnancies are known to come with many mechanical and pathophysiological changes, which necessitates adjustments in immunity in order to integrate the foetus [12]. This means pregnant women generally are more susceptible to infection which could in turn affect the foetus. The significance in primigravida women in this study requires further study. The change in physiology of mothers and more especially for mothers who get pregnant for the first time could predispose neonates to some adverse outcomes such as sepsis. The odds of developing culture proven sepsis among neonates born outside health facilities in this study was 2.29 higher compared to those born within health facilities, though insignificant. This was also observed in a similar study in Uganda [10]. Though other studies have reported birth weight, Apgar score, and sex as risk factors for neonatal sepsis, findings in this study revealed no significant association. This agrees with a similar study in South Korea that suggests an association between defaulting in antenatal clinic attendance and neonatal sepsis [13]. Therefore, care givers, health care providers, and programmes with interests in safe pregnancy and postnatal health need to encourage pregnant women to adhere to antenatal clinic schedules.

## Conclusion

Gram positive organisms were the dominant pathogens causing neonatal sepsis among neonates in this study with *Staphylococcus epidermidis*being the most common. Fewer than recommended antenatal clinic attendance was associated with increased risk of developing neonatal sepsis. Health programmes for maternal and child health should therefore lay emphasis on increased antenatal clinic attendance towards reducing neonatal sepsis.

### What is known about this topic

- Sepsis causes morbidity and mortality in neonates globally;
- Neonatal sepsis occurs within the first 28 days of life;
- Deaths due to neonatal sepsis occur mainly in low- and middle-income countries.

### What this study adds

- Gram positive organism are dominant pathogens causing neonatal sepsis;
- Defaulting in antenatal clinic attendance could increase the risk of neonatal sepsis;
- Primigravida mothers are more likely to have an increased risk of neonatal sepsis.
